# A Dedicated Promoter Drives Constitutive Expression of the Cell-Autonomous Immune Resistance GTPase, Irga6 (IIGP1) in Mouse Liver

**DOI:** 10.1371/journal.pone.0006787

**Published:** 2009-08-26

**Authors:** Jia Zeng, Iana Angelova Parvanova, Jonathan C. Howard

**Affiliations:** Institute for Genetics of the University of Cologne, Cologne, Germany; BMSI-A*STAR, Singapore

## Abstract

**Background:**

In general, immune effector molecules are induced by infection.

**Methodology and Principal Findings:**

However, strong constitutive expression of the cell-autonomous resistance GTPase, Irga6 (IIGP1), was found in mouse liver, contrasting with previous evidence that expression of this protein is exclusively dependent on induction by IFNγ. Constitutive and IFNγ-inducible expression of Irga6 in the liver were shown to be dependent on transcription initiated from two independent untranslated 5′ exons, which splice alternatively into the long exon encoding the full-length protein sequence. Irga6 is expressed constitutively in freshly isolated hepatocytes and is competent in these cells to accumulate on the parasitophorous vacuole membrane of infecting *Toxoplasma gondii* tachyzoites.

**Conclusions and Significance:**

The role of constitutive hepatocyte expression of Irga6 in resistance to parasites invading from the gut via the hepatic portal system is discussed.

## Introduction

In general, immune resistance genes show low rates of constitutive transcription, and rely on signals generated via infection for their transcriptional induction. The most powerful mediator of elevated transcription of resistance genes is interferon (IFN) γ, secreted early in the response to pathogens by natural killer (NK) and NKT cells and later by T helper 1 (T_h_1) and cytotoxic T cells (TC1) of the adaptive immune system [1]. The immunity-related GTPase (IRG) family of GTPases, responsible for cell-autonomous immunity against a variety of intracellular pathogens [2], [3], has seemed to be no exception to this generalization. In our own report of the identification of several IRG proteins [4] we documented strong induction of these proteins in the liver 24 hours after infection with the strongly immunostimulatory pathogen, *Listeria monocytogenes*, and showed that this induction critically depended on the presence of the IFNγ-receptor. Similarly, IRG proteins were strongly induced by IFNγ in tissue culture cell lines [4], [5]. Similar conclusions were reached in a histological study of the BALB/c mouse spleen during the course of infection with *Listeria monocytogenes*, where Irga6 (IIGP1) was seen to be induced in several cell types shortly after infection, but no expression was reported before infection [6]. Nevertheless, there are several reports on constitutive expression of members of the IRG family in mice. Taylor reported constitutive expression of Irgm3 (IGTP) in the thymus [7], with similar results noted for Irgb6 (TGTP), while there have been independent reports of expression of IRG proteins in haemopoietic stem cells [8], [9]. In none of these cases was it clear whether the apparently constitutive expression was in fact due to local IFNγ expression of unknown cause in these lymphomyeloid tissues, rather than to truly IFNγ-independent expression.

In this report we show that Irga6, contrary to earlier conclusions, is in fact strongly and constitutively expressed in mouse liver. Furthermore, this expression is independent of IFN signalling and is driven by a typical hepatocyte-specific promoter upstream of a dedicated 5′-untranslated exon (exon 1B). Irga6 is however, also strongly induced by IFNγ, as correctly reported earlier, and this is due to a second, IFN-specific promoter upstream of a second, dedicated 5′ exon (exon 1A). These results provide a rationalization of our earlier observations on the transcript structure of Irga6, where we noted that the EST database contained clones with identical coding sequences but different 5′-untranslated (5′-UT) sequences consistent with alternative 5′ exon usage, and were able to locate the two corresponding exons upstream of the coding exon (exon 2) [10].

With the exception of Irgc ([10] and Rohde in preparation), all mouse IRG proteins are probably involved in a cell-autonomous resistance mechanism against intracellular pathogens. Irga6 contributes to interferon-induced immunity against *Toxoplasma* gondii since expression of a dominant negative mutant of the protein inhibits interferon-induced resistance to the parasite [11]. Furthermore, two independent mouse strains with disruption or loss of the *Irga6* gene show higher susceptibility to infection with the avirulent ME49 strain of *T. gondii* (unpublished results). We show here that endogenous Irga6, constitutively expressed in hepatocytes, is able to accumulate on the parasitophorous vacuole membrane of infecting ME49 strain *T. gondii*, but is ineffective in initiating the vacuolar rupture typical of complex IFNγ-induced responses in which multiple IRG proteins are expressed at high levels [11]–[14].

## Results

### Constitutive expression of Irga6 in liver

**Figure 1:**
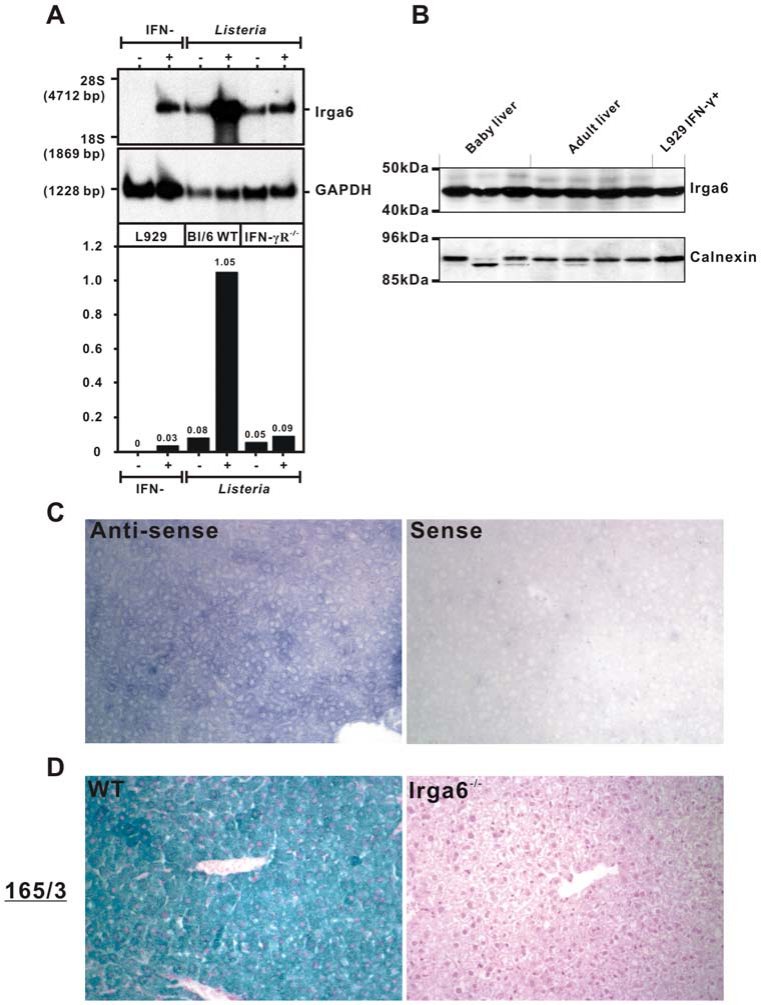
Irga6 is constitutively expressed in healthy mouse liver. A. Northern blot analysis of *Irga6* RNA expression. Total RNA was extracted from livers of wild-type C57BL/6J or IFN-γ receptor knock out (IFN-γR^−/−^) mice, which were either untreated (−) or had been infected with *Listeria monocytogenes* at LD_50_ 24 h earlier (+). The *Irga6* ORF labeled with [α-^32^P]-dCTP was used as probe. L929 mouse fibroblasts, either unstimulated (−) or stimulated (+) with 200 U/ml IFNγ for 24 h, were used as controls. GAPDH was also detected as loading control. The y-axis of the histogram presents relative units of Irga6 signal normalized to GAPDH. B. Western blot analysis of Irga6 protein expression. Detergent lysates of livers prepared as described in Materials and Methods (100 µg/lane) from CB20 baby (3 or 4 days, 3 individuals) and adult mice (4 or 6 weeks, 4 individuals) were probed for Irga6 protein with mAb 10D7. L929 mouse fibroblasts (5,000 cells/lane) stimulated with 200 U/ml IFNγ for 24 h were used as positive control. Calnexin protein was probed as loading control. An endoproteolytic cleavage product of calnexin is apparent in some lanes, especially Lane 2. C. *In situ* hybridization analysis of *Irga6* RNA expression. Livers from adult C57BL/6 mice were fixed, embedded in paraffin and cut into 6 µ sections. DIG-labeled single-stranded RNA sense and anti-sense probes were synthesized by *in vitro* transcription using the 5′ end of the Irga6 ORF (1−490 bp) as template. The hybridized RNA probes were detected with anti-DIG antibody to give a blue stain. For details see Materials and Methods. D. Immunohistochemical analysis of Irga6 protein expression. Paraffin sections of livers from adult C57BL/6 wild-type (WT) and Irga6^−/−^ mice were probed for Irga6 protein with rabbit anti-Irga6 antiserum 165/3. Specific antibody binding was detected histochemically as described in Materials and Methods to yield a green stain. Nuclei were counterstained in red.

When Irga6 was first described [4] we showed by Northern blot that the transcript was massively induced in the liver 24 h after infection with *Listeria monocytogenes*. The autoradiographic exposure of that Northern blot was optimized to obtain the best resolution between transcript levels from uninfected and infected liver, and was not normalized to any other tissue or cell type. The image conveyed the impression that Irga6 is transcribed at very low levels, if at all, in the uninfected liver compared with the infected liver. This is what we believed, and the conclusion was fully compatible with parallel data obtained from tissue culture cells stimulated with IFNγ for 24 h or left unstimulated. Again, the autoradiographic image was optimized to obtain clear resolution between IFNγ-induced and uninduced transcript levels and not normalized to the liver data. We have now revisited that experiment and present a new Northern blot of the same liver RNA pool that was used in the previous study, compared with RNA from IFNγ-induced and uninduced mouse fibroblasts, in this case normalized to GAPDH signal intensity in the two assay systems. The Northern blot and its quantitation are shown in Fig. 1A. The new analysis shows that Irga6 is indeed induced in fibroblasts from a very low expression level, but also shows that the expression level in normal liver from uninfected mice is already even higher than the level reached in IFNγ-induced fibroblasts. This already high level was further enhanced another 10-fold 24 h after L. monocytogenes infection. The elevated expression in liver following L. monocytogenes infection is due to stimulation by IFNγ, since no such effect followed L. monocytogenes infection of IFNγ-receptor-deficient mice. In addition, however, the high basal expression of Irga6 in liver was not significantly affected by loss of the IFNγ receptor, providing a strong indication that basal Irga6 transcription in the liver is not dependent on intrinsic IFNγ stimulation of whatever cause.

The high level of constitutive transcription of Irga6 in the liver was also found to be associated with a high level of Irga6 protein expression in this organ, as shown by Western blot in Fig. 1B. Protein levels relative to calnexin were equal to or higher than those in IFNγ-induced L929 fibroblasts. Constitutive Irga6 protein expression in the liver was also independent of immunological maturity: levels in 1−3 day old mice were no different from levels in mature mice. By *in situ* hybridization (Fig. 1C) and immunohistology (Fig. 1D) the expression of both RNA and protein of Irga6 was confirmed in liver sections, controlled in the case of *in situ* hybridization by the sense probe and in immunohistology on liver sections from an Irga6-deficient mouse derived by gene targeting [15]. The results of both assays confirmed the expression of Irga6 in the liver and both assays also clearly demonstrated that the protein is universally expressed in hepatic parenchymal cells.

### Constitutive expression of Irga6 in other organs

**Figure 2:**
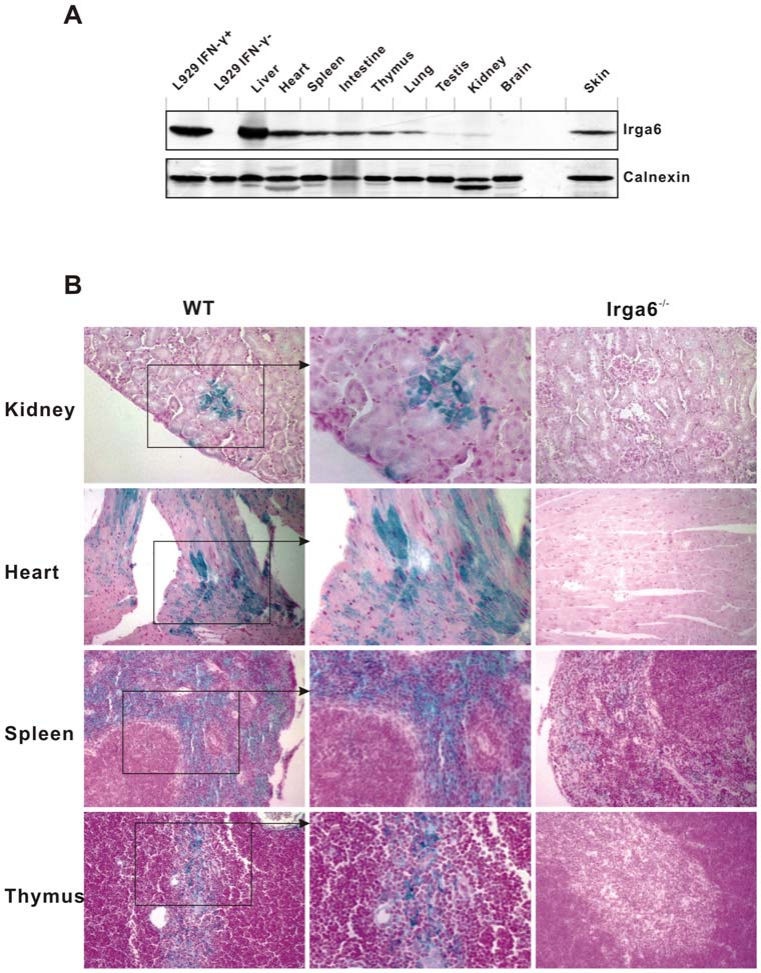
Irga6 is expressed in many mouse tissues. A. Western blot analysis of Irga6 protein expression. Detergent lysates from liver, heart, skin, spleen, intestine, thymus, lung, testis, kidney and brain of wild-type C57BL/6 mice were probed for Irga6 protein using mAb 10D7. Loading for all tissues was 50 µg/lane except for skin, which was 150 µg/lane. L929 mouse fibroblasts (5,000 cells/lane) untreated (L929 IFN-) or stimulated (L929 IFN+) with 200 U/ml IFNγ for 24 h were used as negative and positive controls. Calnexin protein was probed as loading control. An endoproteolytic cleavage product of calnexin is apparent in some lanes, especially in the Heart and Kidney samples. B. Immunohistochemical analysis of Irga6 protein expression. C57BL/6 WT and Irga6^−/−^ adult mouse kidney, heart, spleen and thymus were embedded in paraffin and cut into 6 µ sections. Irga6 protein was probed with mAb 10D7 and detected histochemically to give a green stain. Nuclei were counterstained in red. Frames and arrows show enlarged sections.

**Figure 3:**
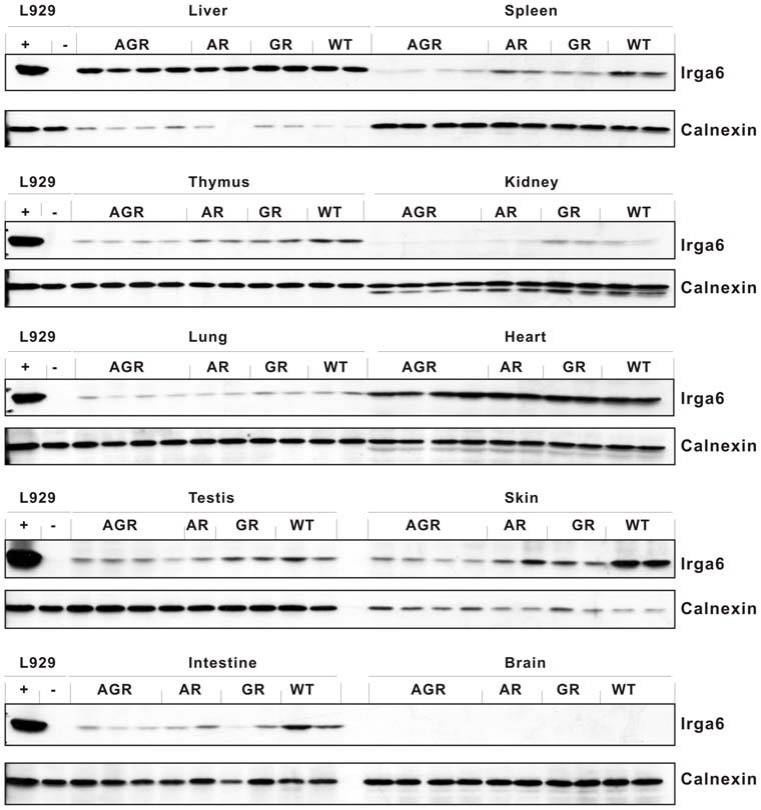
Irga6 expression in different mouse tissues is influenced differentially by IFNs. Western blots for Irga6 protein on detergent lysates of organs from WT 129Sv mice or type I IFN receptor knock-out (AR), type II IFN receptor knock-out (GR), or double knock-out (AGR) mice, on the same genetic background. Blots were probed with mAb 10D7. The organs indicated were taken from 4 individuals for the AGR mice and 2 for the WT, AR and GR mice. The loading for all tissue lysates was 150 µg/lane except for liver, which was 80 µg/lane. L929 mouse fibroblasts (5,000 cells/lane) unstimulated (−) or stimulated (+) with 200 U/ml IFN-γ for 24 h were used as controls. Calnexin protein was probed as loading control.

By Western blot (Fig. 2A) Irga6 is also expressed in untreated mice at widely different levels in other organs, notably heart, skin, spleen and thymus, although nowhere as strongly as in the liver. By immunohistology (Fig. 2B), Irga6 expression was found in some cardiac myocytes but not others. In spleen, Irga6 was expressed exclusively in the red pulp, and in the thymus in the medulla. Surprisingly, an earlier immunohistological analysis of the spleen using a monoclonal antibody (5D9) directed against Irga6 did not report the expression of Irga6 in the red pulp [6]. Occasional patches of Irga6 expression were found in kidney tubular epithelium. No defined compartment expressing Irga6 could be identified in skin despite significant expression in this organ (not shown and Fig. 2A). We considered the possibility that at least some of this apparently constitutive expression of Irga6 might be due to local expression of IFNs, although our observations (Fig. 1) showed this was not the case for the liver. Western blots on organ lysates from single IFNγ-receptor-deficient (IFNGR), single IFN-type I receptor deficient (IFNAR) and double-deficient (IFNAGR) mice were compared with wild type, normalized to calnexin (Fig. 3). As expected, very high constitutive Irga6 expression in the liver was unaffected by single or double IFN receptor deficiency and high expression also persisted in the heart. In skin, spleen, intestine and thymus, however, loss of the IFN receptors markedly reduced the detectable Irga6 signal, with the greatest reduction being in the double receptor deficient mouse. We conclude that Irga6 expression in normal, not intentionally infected, mice arises from two causes, genuinely constitutive expression in liver parenchymal cells and probably also cardiac myocytes, and expression induced by local production of type I and Type II IFNs in skin, spleen and thymus in unidentified cell types.

### Genomic and transcriptional structure of Irga6: two alternative 5′ untranslated exons

The exceptionally high, apparently IFN-independent, expression of Irga6 in liver parenchymal cells suggested that Irga6 transcription in this organ must be under completely different regulation from that typical of fibroblasts and other tissues where interferons appeared to dominate control of Irga6 expression. The genomic organization of the Irga6 gene was reported earlier [10]. In that study we noted that transcription of Irga6 was alternatively initiated upstream of two distinct and widely separated 5′-untranslated exons, which we here name exon 1A and exon 1B respectively for the upstream and downstream exons. These observations suggested that a closer analysis of the 5′ region of the Irga6 gene could be of interest in the present context. Alignments of ESTs from the public databases allowed us to identify Irga6 exons 1A and 1B and infer probable start-points for transcription (Figure S1).

The sequences of exons 1A and 1B diverge considerably in the 5′ region but converge towards near identity close to the splice donor site (Figure S1). Both exons normally splice to the same splice acceptor upstream of the long coding exon 2, 20 bp upstream of the initiator codon (Figure S1). Further short untranslated regions can be found very occasionally introduced between Exon 1A or Exon 1B and the ORF-encoding exon 2 (unpublished results). These unique events are too rare to be dignified by the term “alternative splicing” and too inconsistent to earn the regions concerned the titles of “exons”. A peculiar case recently reported [16] was an Irga6 cDNA carrying both exon 1A and exon1B in tandem with a short spacer between them (NM_001146275). This, till now unique, structure is unlikely to be of any biological significance.

**Figure 4:**
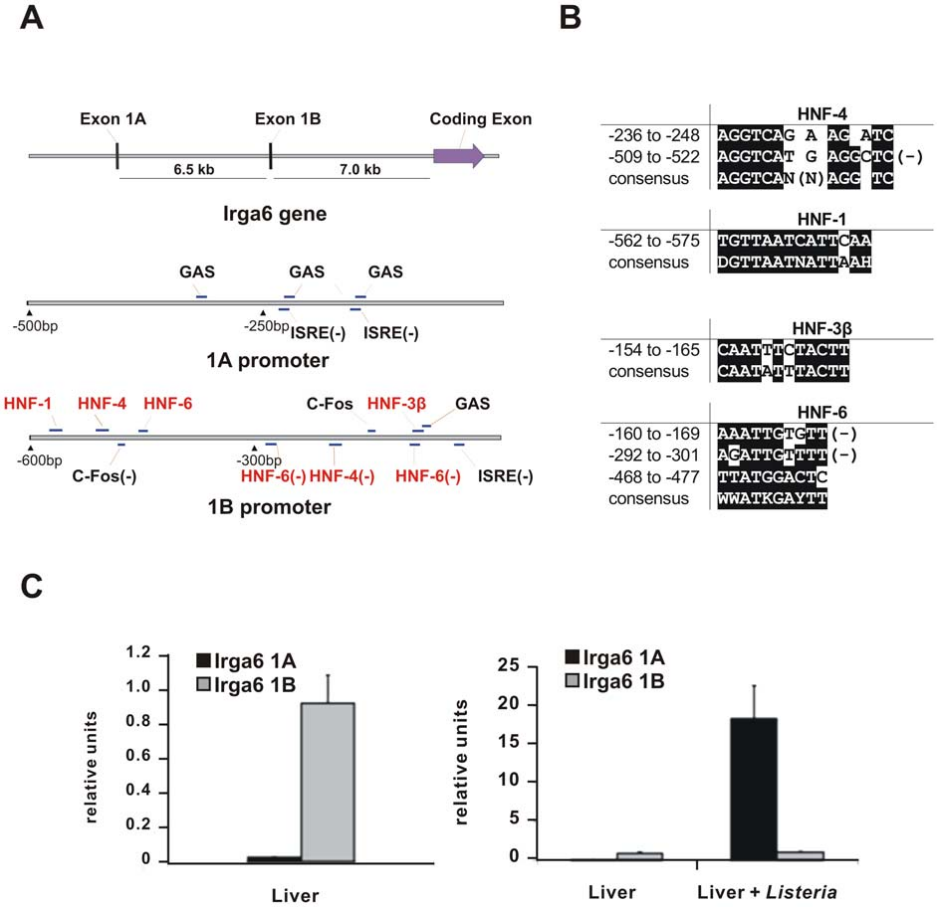
Irga6 constitutive expression in liver uses a different promoter from that used for IFN induction. A. Scheme of *Irga6* gene and promoter structure. The lengths of exons 1A, 1B and the long coding exon 2 are 104 bp, 107 bp and 2233 bp respectively (see also Figure S1). The distance between 1A and 1B is ∼6.5 kb; between 1B and coding exon is ∼7.0 kb (see also Table S2). Putative transcription factor binding sites (GAS, ISRE, C-Fos and HNF) within 500 bp and 600 bp upstream respectively of the Irga6 1A and 1B exons are indicated. Binding sites on the negative strand are marked by (−). B. Putative HNF sites in the *Irga6* 1B promoter. HNF-1 and HNF-3β sites identified as described in Materials and Methods were aligned to the consensus sequences and the position of each sequence upstream of the putative transcription start is given. Bases identical to consensus sequences are shaded in black. Binding sites on the negative strand are marked by (−). D = A/G/T, H = A/C/T, N = A/C/G/T, W = A/T, K = G/T, Y = C/T. C. Real-time PCR analysis of transcript expression of Irga6 carrying the 1A or 1B exons. mRNA from liver of C57BL/6J mice either untreated (Liver) or infected with *Listeria monocytogenes* at LD_50_ for 24 h (Liver+*Listeria*) were subjected to real-time RT-PCR. Relative units here were defined by the expression level of *Irga6* transcript normalized to that of the mouse HPRT. The left hand graph with an expanded scale shows the predominant usage of the 1B promoter in the control liver (Liver). In the right hand graph, the date from the control liver (Liver) is redrawn on the reduced scale necessary to display the enormous increase in transcription of the 1A promoter in the infected liver (Liver+*Listeria*). It is important to take account of the scale difference between the two graphs.

**Figure 5:**
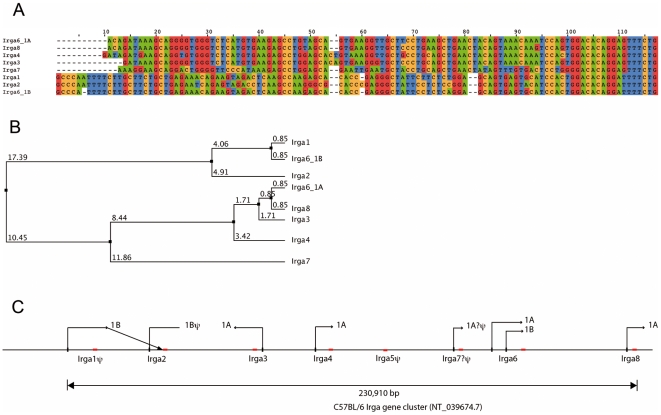
Phylogenetic relationships among the 5′-UT exons of the IRGA genes. A. Alignment of the putative 5′ exons of the IRGA genes. Irga5, which is not listed, is a degraded pseudogene without transcript [10] (see also below). The 5′ exons fall into two sequence classes homologous to the exon 1A and exon 1B of *Irga6* respectively. Exon 1A and 1B of *Irga6* have been presented in detail in Figure S1. Exon 1 of *Irga3*, *Irga4*, and *Irga8* are all inferred from EST sequences and confirmed against the chromosome 18 genomic contig NT_039674.7. The predicted exon 1 of *Irga7* is derived by homology from the other 1A exons. It is positioned and oriented correctly relative to the *Irga7* gene but has not yet been recovered from an EST pool. B. Average distance phylogram based on percentage identity, relating the exon 1 sequences of the IRGA family of genes shown in Fig. 5A above. The numbers recorded above each branch give the percentage difference in sequence along the branch. The distinction between 1A and 1B exons is clear. The predicted exon 1 of Irga7 is an outlier in the 1A group, consistent with the possibility that Irga7 is a pseudogene (see Fig. 5C ). C. Scale map of the IRGA gene cluster on chromosome 18 of the C57BL/6 mouse. The complete sequence domain of the IRGA genes was extracted from the chromosome 18 genomic contig, NT_039674.7. The length of the whole cluster from the 5′ nucleotide of exon 1 of *Irga1* to the 3′ nucleotide of the ORF of *Irga8* is 230,910 nucleotides. In each case, the position of the 5′-UT exon 1 is shown in black and the long exon 2 containing the ORF in red. Arrows above each 5′ exon indicate the direction of transcription. The 3′-UT region of exon 2 is not indicated. The homology group, 1A or 1B (see Fig. 5A and 5B above), is indicated for each 5′-UT exon. The 1B exon upstream of *Irga1* is found associated only with ESTs (e.g. AK149583.1 and AK149556.1) containing the ORF of *Irga2*, indicated by the oblique transcription orientation arrow. The ORF of *Irga1* itself is interrupted by two frame shifts and cannot generate a full-length Irga1 protein. Furthermore, *Irga1* is found in databases exclusively associated with incorrectly-spliced ESTs (e.g. BI658674, BG915086). The transcriptional orientation indicator for Irga2 shows no terminal arrowhead because although the 1B exon of Irga2 is clearly identified by homology no transcripts have been found containing this exon. Irga2 is found exclusively associated with exon 1 of Irga1. *Irga5* was described as a pseudogene earlier [10]. The long exon was identified by homology to exon 2 of other IRGA genes but contains no full-length ORF. No ESTs have been described from *Irga5* and no 5′-UT exon 1 could be identified by homology to either exon 1A or 1B. *Irga7* encodes a full-length ORF in exon 2, and the 5′-UT was readily identifiable by homology. However no ESTs encoding *Irga7* have been found and no transcripts corresponding to *Irga7* were detected in IFN-stimulated L929 fibroblasts (Bekpen, 2005). Thus *Irga7* is probably a pseudogene in the C57BL/6 mouse and in L929 (C3H/An strain) fibroblasts.

Fig. 4A shows diagrammatically the intron-exon structure of the Irga6 gene and the organization of the 1A and 1B promoters (see also Fig. 5 and Table S2). In the genomic structure of Irga6 exons 1A and 1B are separated by 6558 bp on chromosome 18 and the long coding exon 2 begins 7018 bp downstream of the 3′ margin of exon 1B. Definition of the transcriptional start sites for exons 1A and 1B (Figure S1, see also above) allowed for the identification of putative transcription factor binding sites. In our previous analysis 5 IFN response elements were found upstream of exon 1A, including 2 ISREs and 3 GAS sequences, and single GAS and ISRE sequences upstream of exon 1B (Bekpen et al, 2005). A search for liver specific response elements in the 1000 bases upstream of exons 1A and 1B now revealed that exon 1B is preceded by an apparently canonical liver-specific promoter, while no liver response elements were found closer than 900 bases upstream of exon 1A (Fig. 4A). Alignments of the 7 hepatocyte nuclear factor (HNF) promoter elements found in the 1B promoter with the consensus sequence for each element are shown in Fig. 4B. Not only the identity of the HNF elements, but also their array on the putative promoter conform to the pattern of typical liver-specific promoters [17], [18].

To identify the promoter usage responsible for the constitutive and IFN-inducible Irga6 in liver, two primer pairs were designed with 5′ primers specific for exon 1A and exon 1B respectively, and a common 3′ primer in exon 2 (see Materials and Methods) for use in quantitative real-time PCR on cDNA from uninfected and *L. monocytogenes*-infected mouse livers. The result (Fig. 4C), expressed in relative units, showed that essentially all the Irga6 transcripts in uninfected liver were initiated from the 1B promoter, while essentially all of the Irga6 transcripts in *L. monocytogenes*-infected liver were initiated from the 1A promoter. The relative expression of the 1A-derived transcript was increased approximately 800-fold by *L. monocytogenes* infection. Perhaps unexpectedly, the expression of the 1B-derived transcript was apparently not noticeably affected by the enormous increase in transcription from the 1A promoter. Indeed, a marginal increase was measured.

A differential PCR analysis of the use of the 1A and 1B promoters for constitutive Irga6 expression in other organs of uninfected animals showed extremely low transcript levels compared with liver, and no consistent organ-specific preference for the 1A or 1B promoters (Fig S2). Unexpectedly, constitutive expression of Irga6 in the heart was not restricted to use of the 1B promoter, thus the explanation for this constitutive expression is clearly different, and would justify an intensive analysis.

Exon1B, associated with the hepatocyte-specific Irga6 promoter, has homologues in the 5′ regions of two other genes of the IRGA cluster on mouse chromosome 18. Irga1 and Irga2, whose coding sequences cluster phylogenetically with Irga6 [10], both possess potential 5′ exons with strong sequence homology to exon 1B of Irga6 (Fig. 5A and B). In the case of Irga1 the homology to the exon 1B region of Irga6 continues upstream across the whole region populated by HNF binding sites, while in Irga2 only the distal cluster of HNF binding sites is conserved (Table S1). Both Irga1 and Irga2 are probably functionally pseudogenes [10]. For unknown reasons, Irga1 fails to splice normally from the 5′-UT to the 3′ coding exon, and also has two short frame-shifting deletions in exon 2 which would lead to an abnormal truncated protein. Irga2 has a surprising splicing arrangement, using exclusively the 5′-UT exon of Irga1 rather than its own 5′ exon (which is identified in Fig. 5A by homology), and then leapfrogging the Irga1 coding exon and the entire intergenic region before splicing correctly into the long coding exon 2 of Irga2. The products of this unusual splicing arrangement are apparently complete, but we have till now been unable to detect an Irga2 protein product in IFNγ-induced cells derived from Irga6^−/−^ fibroblasts using a rabbit anti-Irga6 antiserum that cross-reacts strongly on bacterially-expressed Irga2 (unpublished results). Irga2 transcripts of this type are strongly induced by IFNγ, presumably from the ISRE and GAS sites located in the Irga1 exon 1 promoter [10]. The reason for the absence of an Irga2 protein product *in vivo* is not known. The bottom line, however, is that Irga6 is the only functional IRGA protein expressed from its constitutive liver-specific 1B promoter. The remaining genes in the Irga cluster have 5′ untranslated exons homologous to the 1A exon of Irga6, preceded by clusters of canonical interferon-inducible elements ([10] and Fig. 5A alignment). The complete exon organization of the Irga cluster on chromosome 18 is given in Fig. 5C, along with a detailed description

### Expression of Irga6 in isolated hepatocytes

**Figure 6:**
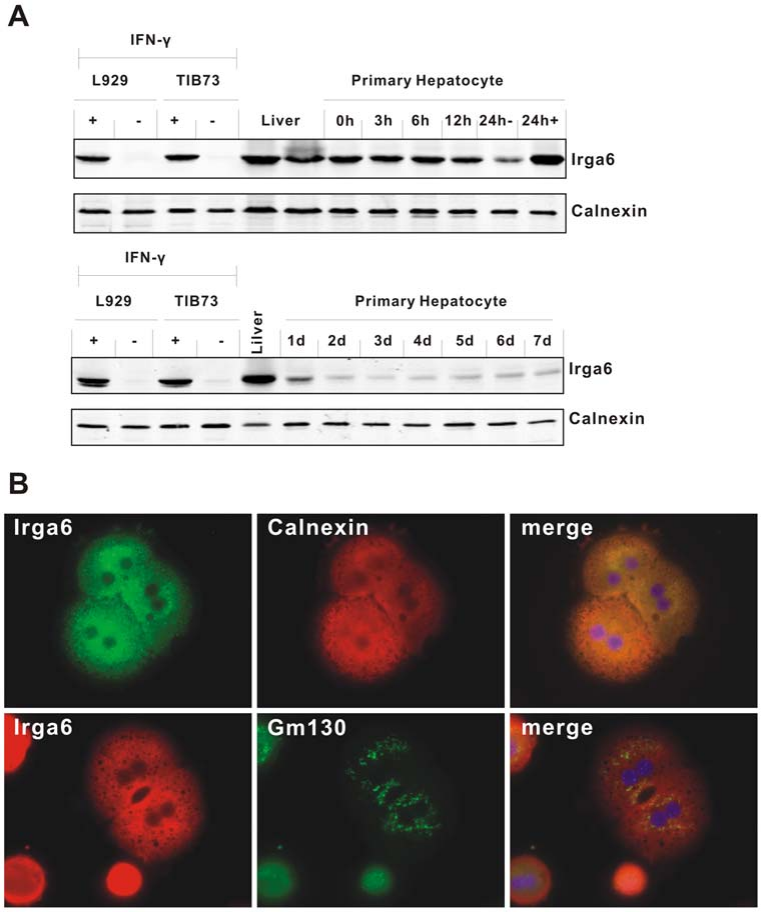
Constitutive and induced expression of Irga6 in mouse primary hepatocytes. A. Western blot analysis of Irga6 protein expression. C57BL/6 mouse primary hepatocytes were isolated as described in Materials and Methods. Protein lysates of liver or primary hepatocytes cultured and harvested at indicated hours (h) or days (d) post-isolation were probed for Irga6 protein with mAb 10D7 by Western blotting. Hepatocytes were cultivated with (24h+) or without (24h-) 200 U/ml IFN© for 24 hours. L929 and TIB73 cells (5,000 cells/lane) stimulated with IFNγ (200 U/ml) for 24 h were used as positive control. Calnexin protein was probed as loading control. B. Immunofluorescence analysis of Irga6 protein expression in hepatocytes. C57BL/6 mouse primary hepatocytes were isolated, cultured for 6 h, fixed and immunostained for Irga6 protein. Double staining using either Irga6 mAb 10E7 (green) and calnexin rabbit antiserum (red) (upper row) or Irga6 rabbit antiserum 165/3 (red) and anti-GM130 mAb (green) (lower row) were performed. Nuclei were counterstained with DAPI (blue).

To confirm the expression of Irga6 in hepatocytes, a number of *in vitro* established cell lines of hepatic origin were assayed for constitutive Irga6 expression. Very low levels of Irga6 were detected, while in all cases Irga6 could be induced with IFNγ (Fig. 6A and data not shown). Thus the elevated constitutive expression of Irga6 found *in vivo* appeared to reflect a differentiated property of hepatocytes that, in common with other differentiated functions of these cells, is lost on *in vitro* culture [19]–[21]. This could be directly confirmed by cultivation of primary hepatocytes isolated from mouse liver. The Western blot shown in Fig. 6A (upper panel) shows that expression of Irga6 in isolated primary hepatocytes is higher than the calnexin control signal. This intensity of expression is maintained up to 6 h of culture but thereafter drops markedly. The decline in expression at 24 h can, however, be prevented and indeed reversed if the hepatocytes are cultured for this period with IFNγ. The expression of Irga6 in primary hepatocytes reached a minimum by 2 days of culture and remained essentially stable at this low level for up to 5 days thereafter (Fig. 6A, lower panel). The residual expression level after 2 or more days of hepatocyte culture was only slightly higher than that seen in an unstimulated line of hepatocyte-derived tissue culture cells, TIB73. Other IRG proteins were also detected above background in primary hepatocytes. The levels of Irgm1, Irgb6 (Fig S3) and Irgd (not shown) were significant but below the expression levels in IFNg-induced fibroblasts. Irgm3 was barely detectable above background (Fig S3 and data not shown). All of these proteins were strongly inducible in primary hepatocytes by IFNg (Fig S3 and data not shown)

Immunofluorescence analysis of the expression of Irga6 in freshly isolated primary hepatocytes confirmed the high level of expression (Fig. 6B). The Irga6 signal accurately co-localised with the signal for the ER protein, calnexin. No significant expression of hepatocyte Irga6 was apparently associated with the distributed Golgi elements of these cells.

### Accumulation of Irga6 on the parasitophorous vacuoles of T. *gondii* infecting isolated primary hepatocytes

**Figure 7:**
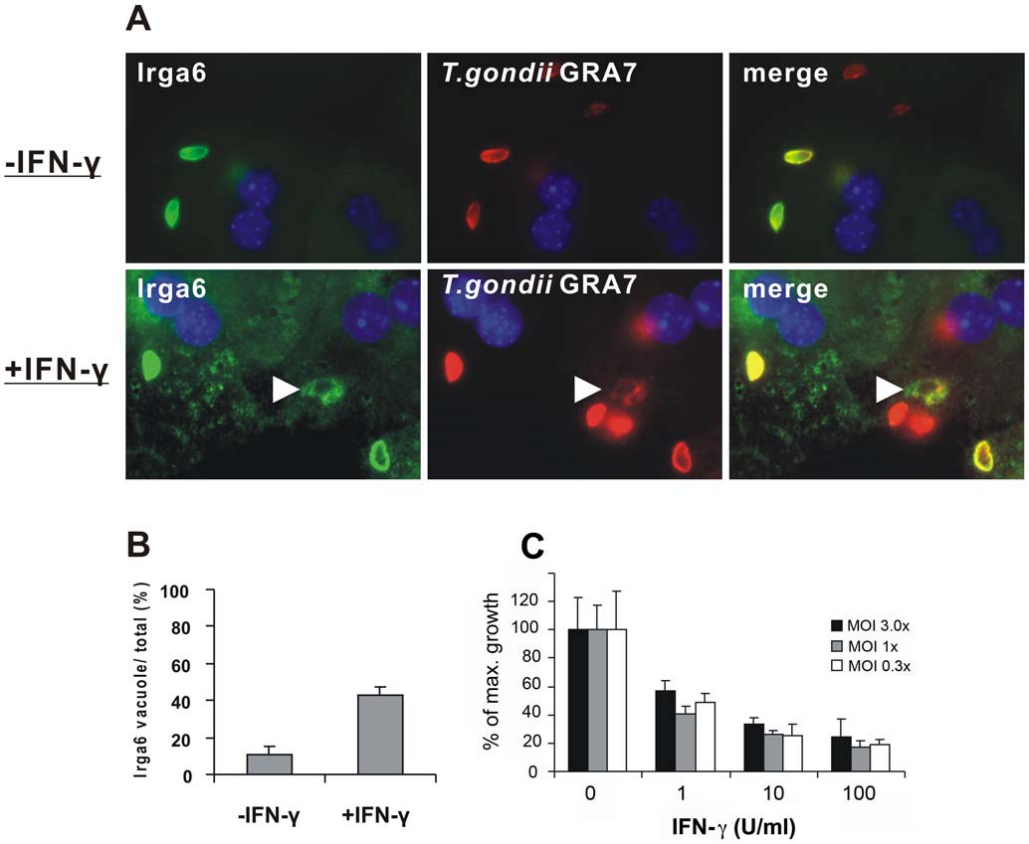
Constitutive and IFNγ stimulated Irga6 proteins accumulate around *T. gondii* PVs in mouse primary hepatocytes. A. Immunofluorescence analysis of Irga6 protein accumulation at *T. gondii* vacuoles in murine primary hepatocytes. C57BL/6 murine primary hepatocytes were freshly isolated and either stimulated (+IFNγ) for 12 h with 200 U/ml IFNγ or left untreated and cultured for 3 hours (-IFN-γ). The cells were inoculated with *T. gondii* (strain ME49) at a MOI of 3 for 2 h, fixed and immunostained. Double staining for Irga6 with mAb 10E7 (green) and GRA7 with anti-GRA7 mAb (red) was performed. Nuclei were counterstained with DAPI (blue). White arrowhead indicates a disrupting vacuole. B. Quantification of Irga6 positive *T. gondii* PVs in the experimental setup described in (A). Percentages of Irga6 positive PVs per total intracellular *T. gondii* vacuoles are given. Intracellular parasites were identified by the distinct GRA7 pattern. The means of two or three independent experiments

As previously reported, Irga6 and other IRG proteins accumulate rapidly on the parasitophorous vacuoles of infecting T. *gondii* in IFNγ-induced cells [11], [22]. We were interested to know whether the constitutively expressed Irga6 in primary hepatocytes could also accumulate on the T. *gondii* PV. Freshly isolated primary hepatocytes were therefore infected with T. gondii strain ME49 and Irga6 distribution was examined by immunofluorescence. Intense ring-shaped fluorescence of Irga6 surrounding some infecting T.gondii was observed (Fig. 7A). It was, however, also noted that the proportion of Irga6-coated T. gondii vacuoles, at 10%, was much lower than previously observed in IFNγ-induced fibroblasts and other cells, which is normally in the range from 50% to 70% [11]. In unstimulated primary hepatocytes Irga6 was the only IRG protein that was detected on the T. gondii PVM. Irgb6 and Irgd could not be detected at the PVM despite their significant basal expression levels (data not shown). In IFNγ-stimulated primary hepatocytes, however, the normal pattern of PVM loading with these IRG proteins was observed, and the typical quantitative hierarchy of loading (Irgb6>Irga6>Irgd>Irgm3) described in detail elsewhere (Khaminets et al, in preparation), was restored (not shown). In IFNγ-treated cells, T. gondii PVs coated with IRG proteins became disrupted during the first few hours after infection [11], [14]. No disruption of Irga6-coated T. gondii PVs was observed in primary hepatocytes without IFNγ treatment, but disrupted vacuoles were readily seen in infected IFNγ-induced primary hepatocytes (Fig. 7A). IFNg-induced primary hepatocytes were also able to control T. gondii replication (Fig. 7C).

## Discussion

We have shown that Irga6, an IFN-inducible member of the immunity-related IRG protein family in the C57BL/6 mouse, is also expressed constitutively and strongly in normal mouse liver through use of a dedicated, liver-specific promoter. IFN-inducible and constitutive expression are initiated from separate promoters. Each promoter is upstream of a dedicated 5′ exon that splices into the single long protein-coding exon 2 just upstream of the translation start. Thus the proteins derived from the two promoters are identical. Inducibility by interferons is a strong indication for a role in induced resistance to infection [1], and the strong IFN inducibility of Irga6 and all the other mouse IRG proteins except Irgc seems to correlate with their role in countering infections by exogenous intracellular parasites [3]. The high constitutive expression of Irga6 in liver stands in contrast to that principle and presents something of a paradox. Certainly, constitutive expression of an early-acting resistance mechanism in barrier tissues may be considered preadaptive, and it was notable that a significant level of Irga6 expression was observed in the skin, although it could not be localised histologically to a specific cell layer and was in any case substantially lower than the liver-specific expression. Furthermore the apparent partial dependence of skin-specific Irga6 expression on IFN action implied by the reduced expression in IFN receptor-deficient mice may suggest that Irga6 expression in the skin is not primarily constitutive but rather due to continuous low-level stimulation of immunity in this barrier site. Low, IFN-dependent expression of Irga6 in lymphoid organs such as spleen and thymus presumably reflects low-level immune-related activity. Histologically, expression in red-pulp of spleen and in medulla of thymus suggests that macrophages may be the main sites of elevated Irga6 expression though this has not been investigated directly.

However these considerations do not help to explain the genuinely constitutive expression of Irga6 at very high levels in liver parenchymal cells. Here, expression relative to the calnexin control was higher than in IFN-induced fibroblasts, both at the mRNA and protein levels, and was not detectably reduced by loss of either or both the type I and type II IFN receptors. The near exclusive use of the hepatocyte-specific 1B promoter for IFN-independent expression further confirms that this expression is indeed truly constitutive.

To rationalize constitutive Irga6 expression in the liver, it may be argued that the liver is in some sense a barrier organ for pathogens such as *L. monocytogenes* and *Plasmodium* that above all favour liver parenchymal cells for their initial proliferative episode. Liver is an early site of infection in peroral *T. gondii* infection after dissemination from the wall of the intestine ([23] and Oliver Liesenfeld, personal communication) presumably via the hepatic portal system. However expression of Irga6 at high levels in hepatocytes in the absence of IFNγ, as shown here, raises a further complex issue. We have shown elsewhere that both the resting localisation of Irga6 in cells and its ability to accumulate normally on the *T. gondii* parasitophorous vacuole is dependent on co-expression of multiple IFN-inducible members of the IRG protein family ([24]; Khaminets et al, in prep). At first sight, Irga6 expressed in resting hepatocytes appears to break this rule. The protein is accurately localised on the endoplasmic reticulum, and is able to accumulate on the *T. gondii* PVM. However the number of vacuoles showing detectable Irga6 accumulation is abnormally low, a number that is substantially restored towards expected levels by prior induction with IFNγ. Furthermore, in the absence of IFNγ induction, no disruption of the *T. gondii* vacuole was detected, suggesting that a high level of expression of Irga6 alone is not sufficient to complete the resistance mechanism. We considered the possibility that other IRG proteins may also be expressed at measurable levels in hepatocytes, even if not at the level of Irga6, thus enabling localisation of Irga6 to the ER and partially enabling access to the *T. gondii* PVM. Irgb6, Irgm1 and Irgd were all expressed at detectable though still relatively low levels. The striking exception is Irgm3, which was essentially undetectable in unstimulated primary hepatocytes but was strongly induced by IFNg. Interestingly, Irgm3 is expressed constitutively in whole liver, but is associated exclusively with scattered, much smaller cells probably associated with the sinusoids. These cells are lost during the hepatocyte purification. We reported inefficient Irga6 loading of the T. gondii PVM earlier [24] in cells in which Irga6 alone of the effector IRG (GKS subfamily) proteins was co-expressed with the three regulator proteins, Irgm1, Irgm2 and Irgm3 [24]. We further showed that expression of all 3 Irgm proteins was required for loading. We have since shown that quantitatively normal PVM loading of Irga6 in such reconstruction experiments can be achieved by addition of normal levels of Irgb6 and/or Irgd (Khaminets et al, in prep). The anomalies of IRG loading in unstimulated hepatocytes may be therefore be attributable to the apparently complete absence of Irgm3 and the relatively low levels of other IRG proteins. Thus hepatocytes constitutively express an “unbalanced” collection of IRG proteins that supports only inefficient loading of the *T. gondii* PVM. Evidently, the complement of highly expressed IRG proteins would normalize in hepatocytes as soon as the IFNγ levels begin to rise during infection, and we show that IFNg-induced primary hepatocytes are able to control T. gondii replication (Fig S3). Whether early expression of Irga6 alone accelerates the development of full cell-autonomous resistance is unclear. An adaptive explanation for the high and dedicated expression of Irga6 in hepatic parenchymal cells is therefore not yet available.

An unexpected precedent exists for the complex regulation of expression of Irga6. The human and mouse RNA-specific adenosine deaminase, ADAR1, which is a classical Type I interferon-inducible gene associated with the dsRNA recognition and degradation pathway, is also transcribed from two distinct promoters, named exon 1A and exon 1B, each subtending its own untranslated 5′ exon [25]–[27]. Like Irga6, one of the promoters is interferon-inducible while the other is expressed constitutively, in this case in embryos and in the adult brain. Despite the striking similarities there are also differences between the two situations. The two Irga6 promoters regulate the synthesis of identical protein products. The two ADAR promoters, on the other hand, control the expression of distinct products since the 1A exon behind the interferon-inducible promoter also contains an initiator methionine, while the constitutively expressed 1B exon does not and protein translation is initiated from a second methionine codon in exon 2, generating a shorter protein. Furthermore, there is evidence that the RNA editing activity of ADAR may be exercised on host cell mRNA, and that the constitutive form of ADAR1 is necessary for normal embryonic development. In contrast, mice carrying a genomic disruption in the Irga6 protein coding region show no developmental abnormalities and are perfectly viable and fertile, while showing a significant susceptibility to infection with an avirulent strain of *T. gondii* (unpublished results and Liesenfeld, O, personal communication). It is not excluded that the constitutive expression of Irga6 in the liver may contribute to a pathogen resistance function, but our data show that the high constitutive expression of Irga6 leads only to limited loading onto the *T. gondii* parasitophorous vacuole membrane and is unable to lead to disruption of the vacuolar membrane. For the time being, therefore, the functional meaning of constitutive expression of Irga6 in mouse liver remains to be identified.

## Materials and Methods

### Mice

Specific pathogen-free C57BL/6 and CB20 mice were obtained from the mouse facility in the Institute for Genetics, University of Cologne. In Irga6-deficient (Irga6^−/−^) mice the entire coding exon (exon 2) of the Irga6 gene was removed from the C57BL/6 genome by Cre-mediated recombination ([15] and unpublished results). Type I, Type II and double IFN receptor deficient mice (IFNAR [28], IFNGR ([29], IFNAGR [30]) on the 129SV background; were kindly provided by Dr. Marina Freudenberg (Max Planck Institute for Immunobiology, Freiburg and Professor Hans Hengartner (Institute for Experimental Immunology, University of Zürich). RNA preparations from the livers of C57BL/6 and IFNGR-deficient mice infected i.p. with *Listeria monocytogenes* strain EGD at 50% of the lethal dose were described earlier [4].

Experiments involving mice were performed following the guidelines given in Licence Nr. 50.203.2-K 13, 21/02 “Cell-biological basis of innate immunity” issued by the administrative authority of the City of Cologne.

### Serological reagents

Primary serological reagents used were: anti-Irga6 165 rabbit antiserum (ref), anti-Irga6 mouse monoclonal antibodies (mAb) 10D7 and 10E7 (gifts from Jens Zerrahn, MPI for Infection Biology, Berlin), anti-calnexin rabbit antiserum (Biomol, Hamburg), anti-Gm130 mAb (BD Transduction Laboratory), anti-GRA7 5–241–178 mouse mAb (gift from R. Ziemann, Abbott Laboratories). Secondary fluorescent antibodies were goat anti-mouse Alexa 546/488, goat anti-rabbit Alexa 546/488 (Molecular Probes).

### Northern blot analysis

5 µg of total RNA from liver or cells was separated on 1% agarose/formaldehyde gels and transferred onto Hybond-N+ membrane (Amersham Biosciences) by standard procedures. The blots were probed with randomly primed [α-^32^P]dCTP-labeled Irga6 ORF generated with the Rediprime DNA labeling system (Amersham Life Sciences). The similarly labeled ORF of mouse glyceraldehyde-3-phosphate dehydrogenase (GAPDH) was used as a control probe to reveal the amount of loaded total RNA, and the RNA Millenium Marker (Ambion) was used as a RNA size standard. Hybridizations were performed overnight at 42°C in a buffer containing 50% formamide, 5×Denhardt's solution (100 mg/ml each of Ficoll400, polvinylpyrrolidone and BSA Cohn Fraction V), 5×standard saline-phosphate-EDTA (SSPE), 1% SDS, and 10% dextran sulfate. Membranes were washed under stringent conditions. The hybridization signal was detected by autoradiography, using Kodak X-OMAT AR films.

### Protein extraction and Western detection

Mouse tissues or cells were snap frozen in liquid nitrogen, ground fine in a pestle and mortar (only for tissue), weighed and lysed in the following buffer (150 mM NaCl, 50 mM Tris, 1% NP-40, 0.1% SDS, 0.5% deoxycholate, 5 mM EDTA, 1% Triton X-100, pH 8.0) with protein inhibitor cocktail (Complete Mini, Roche) to a standard final concentration. The lysates were homogenized by repeated passage through a 20G syringe needle and cleared by centrifugation (30 min, 23,000 g). The supernatants were then subjected to standard western blot analysis.

### Histology

#### Tissue preparation

Mouse tissues were fixed in TBS/4% paraformaldehyde at 4°C and dehydrated through an ethanol series at 4°C (50%, 70%, 90%, 96%). Tissues were then transferred into isopropanol and finally into paraffin∶isopropanol (1∶1) solution at 60°C. The isopropanol was evaporated and fresh paraffin was then replenished several times at 60°C before the sections were moved to room temperature (RT). The embedded tissues were cut with a microtome RM 2065 (Leica Microsystems) into 6 µm thick serial sections.

#### Immunohistochemistry

Paraffin sections were de-waxed in xylene and post-fixed in 4% paraformaldehyde for 1 h at RT. Protein epitopes were then demasked by boiling for 10 min in 10 mM citrate buffer, pH 6.0. Non-specific protein binding and endogenous peroxidases were saturated with quenching buffer (1% BSA, 0.3% H_2_O_2_ in PBS) for 20 mins. After PBS washing, the sections were probed with 165/3 antiserum in DAKO diluent (DAKO, Hamburg). The HRP colour reaction was performed with the peroxidase substrate kit HistoGreen (Linaris). The nuclei were counterstained with Nuclear Fast Red.

#### *In situ* hybridization

The Irga6 DNA template for the RNA probe were amplified using antisense (5′-CGGGGAAGTCATAGTGACAA-3′) and sense primers (5′-GGATCAGGGAAGTCCAGCTT-3′) and cloned into the vector pGEM-T-easy (Promega). The RNA-probes were synthesized using Sp6- or T7-RNA-polymerase and labelled with Digoxigenin (DIG) using the DIG labelling reaction mix (Roche). After de-waxing and post-fixation, paraffin embedded sections of mice liver were digested with 10 µg/ml Proteinase K in 0.1 M Tris pH 7.5 for 10 min at 37°C. The positively charged amino acids were blocked by 0.1 M triethanolamine pH 8.0, 0.25% acetic acid anhydride. Hybridization was performed overnight at 70°C in a buffer containing 50% formamide, 5×SSC pH 7.0, 1×Denhardt's solution, 0.1% Tween20 and 0.1 mg/ml yeast tRNA (Roche). The slides were washed under stringent conditions. The hybridization was detected by probing with anti-DIG Fab fragments conjugated to alkaline phosphatase (Roche) using BM purple substrate (Roche) according to manufacturer's instruction.

### Cell culture

#### Established cell lines

L929 mouse fibroblasts, and TIB-75 hepatocytes were cultured in Iscove's Modified Dulbecco's Medium (IMDM)supplemented with 10% FCS (Sigma-Aldrich, Deisenhofen, Germany), 2 mM L-glutamine, 1 mM sodium pyruvate, 100 U/ml penicillin, and 100 µg/ml streptomycin. Cells were induced with mouse IFN-γ (Cell Concepts) at a concentration of 200 U/ml for 24 h.

#### Murine primary hepatocytes

Hepatocytes were isolated from liver using a two-step liver perfusion method (modified from [31]). Mice were anaesthetized with 2–5 ml Avertin and injected with 100 µl heparin-Na (5000 IU/ml). Mice were dissected and the liver portal vein was canulated. The liver was first perfused with pre-warmed (37°C) Krebs-Ringer-Buffer (KRB) (115 mM NaCl, 5.9 mM KCl, 1.2 mM MgCl_2_, 1.2 mM NaH_2_PO_4_, 1.2 mM Na_2_SO_4_, 2.5 mM CaCl_2_, 25 mM NaHCO_3_, 10 mM glucose) at a flow rate of 3 ml/min for 30 min, then with pre-warmed 50 ml KRB with 1 mM EDTA, and finally with 100 ml KRB with 0.5 mM CaCl_2_ and 0.05% collagenase (SIGMA) at the same flow rate. Hepatocytes were liberated from the liver capsule and filtered through 100 µm gauze. The cell suspension was washed twice at 4°C with washing buffer (1xHanks buffer, 0.5% BSA, 10 mM HEPES pH 7.65) at low speed (50 g) and then twice with DMEM/Ham's F12 1∶1 Medium (Supplemented with 10% FCS, insulin, transferrin and selenium (ITS, Roche) at 1%, 0.1 µM dexamethasone and 100 U/ml Penicillin/100 µg/ml streptomycin buffered by 15 mM HEPES). Cells were then pre-seeded for 15 min on collagen coated plates to eliminate tightly adherent cells and then transferred to other collagen coated plates for culture for several days. Note: All buffers and gauze were sterilized and great care was taken when handing the cells to avoid high mortality.

### Immunofluorescence procedures

Immunofluorescence of cultured cells was performed as described previously [32] and examined with an Axioplan II fluorescence microscope equipped with an AxioCam MRm camera using the Axiovision 4.7 software (all Zeiss).

Cells were fixed in 3% paraformaldehyde and permeabilized with 0.1% saponin and blocked with 3% BSA in PBS (Roth). The cells were analyzed using a Zeiss (Oberkochen, Germany) Axioplan II fluorescence microscope equipped with a cooled charge-coupled device camera (Quantix; Photometrix, Tucson, AZ).

### Procedures involving *Toxoplasma gondii*

Tachyzoites from *T. gondii* strain ME49 were maintained by serial passage in confluent monolayers of human foreskin fibroblasts (HS27, ATCC) as described previously [11]. Mouse primary hepatocytes were either left untreated or stimulated with IFN-γ (R&D Systems) at 200 U/ml for 24 h prior to infection. For immunostaining, hepatocytes were inoculated with *T. gondii* at a MOI of 3 for 2 h. Extracellular parasites were then removed by extensive washing with PBS and cells were processed for immunostaining as above. Vacuoles containing intracellular parasites were identified by immunostaining for the *T. gondii* dense granule protein GRA7 that is associated with the intravacuolar network and the PV membrane after infection (reference).

### Bioinformatics

Public databases (NCBI (http://www.ncbi.nlm.nih.gov) and ENSEMBL (http://www.ensembl.org)) were screened for Irga6 with the Basic Local Alignment Search Tool, BLAST [33] to identify ESTs and cDNAs that could clarify Irga6 5′ exon usage. The recovered sequences were aligned to distinguish ESTs based on Exon 1A and exon 1B and to identify the splice site between exon 1A or 1B and exon 2. Sequence alignments were performed either with Vector NTI (InVitrogen), or using ClustalW2 at the EBI-Sanger website (http://www.ebi.ac.uk/Tools/clustalw2/) and edited manually. Shading of alignments was conducted with GeneDoc (Version 2.6.002) (http://www.nrbsc.org) or JalView (http://www.jalview.org/). Transcriptional start points for exon 1A and exon 1B were defined informally by the following criteria and being the 5′ base of the longest 5′ extensions, confirmed by a second sequence and present in the genomic sequence. Since the genomic sequence standard for comparison was based on the C57BL/6 genomic sequence for chromosome 18 (contig NT*_039674.*7), cDNA sequences derived from C57BL/6 were chosen to define the transcriptional start although longer plausible 5′ extensions were found in ESTs from other mouse strains. The regions up to 1000 bp upstream of the transcription start point were designated as the putative promoter regions for each exon 1 of Irga6 and screened for transcription factor binding sites. ISRE (IFN-stimulated response element), GAS (gamma-activated sequence), and hepatocyte nuclear factors HNF1 and HNF3 [34] binding sites were detected with CONSITE (http://asp.ii.uib.no:8090/cgi-bin/CONSITE/consite/, [35] and were confirmed manually. HNF4 and HNF6 binding sites were identified by manual comparison to published consensus binding sequences [36]–[39].

### Real-time PCR

Real-time quantitative polymerase chain reaction (qPCR) analysis was performed in a Light Cycler I System using a LightCycler SYBR Green I PCR kit (both Roche). The 5′ primers specific for Irga6 exon 1A (5′-TGCTTCCTGAAGCTGAACTA-3′) and 1B exon (5′-ACCGAGGGCTATTCCTCTCA-3′) together with a common 3′ primer for the coding exon (5′-CAGAGAAGGGATGATATTCAC-3′) were used to detect and distinguish Irga6 transcripts containing exons 1A and 1B. Hypoxanthine-guanine phosphoribosyltransferase (HPRT) specific primers (5′ primer 5′-ATTAGCGATGATGAACCAGG-3′, 3′ primer 5′-TGGCCTATAGGCTCATAGTG-3′) were used to provide an input control. cDNA synthesized from mRNA extracted from liver was used as PCR template The level of Irga6 transcript was normalized to that of HPRT in the same sample. Melting curve analysis was performed after each run. Each sample was tested in duplicate or triplicate. The quantitation of all transcripts was achieved by comparison to an external standard based on five serial tenfold dilutions of a pGEMTeasy plasmid containing a full-length construct of Irga6 carrying the 5′UT exon 1A; the dilutions ranged from 10^6^ to 10^2^ plasmid copies per assay.

## Supporting Information

Table S1 Promoter HNF elements
(0.03 MB DOC)

Table S2 Directory of Irga gene sequence elements on chromosome 18 genomic contig, NT_039674.7
(0.06 MB DOC)

Figure S1 Mouse EST sequences corresponding to Irga6 were recovered from the public databases using Megablast. The figure shows an alignment of all ESTs with extended sequence in the 5′-untranslated region. The genomic regions corresponding to exon1A and exon 1B were identified from the C57BL/6 chromosome 18 contig, NT_039674.7, and are given at top and bottom of the alignments. The splice boundary between both exon 1A and exon 1B and exon 2 is indicated by arrows above and below the alignment. The last three bases of each EST sequence in the alignment form the initial start codon of the Irga6 translated protein. We used the 5′ nucleotide of the two C57BL/6-derived ESTs, AI116617 and AI662996, to define the start-point of transcription for exon 1A and exon 1B respectively. These correspond to nucleotide 57,535,681 of NT_039674.7 and for exon 1A and nucleotide 57,542342 for exon 1B (both printed in red). Non-genomic 5′ sequence extensions were removed from some EST sequences before alignment. A splice variant of Irga6 was described recently in IFN-induced mouse cells which carries both exon1A and exon 1B in tandem in that order, with a short linker sequence between them (NM_001146275) [16]. This is a unique case and undoubtedly should not be considered a normal splice variant. * indicates mouse strain of origin of EST. ** indicates ESTs from libraries where the Mus domesticus strain of origin was not recorded.
(0.26 MB TIF)

Figure S2 Real-time PCR to analyze Irga6 expression in RNA purified from different organs of adult C57/BL6 mice. Results are presented as means with standard deviations from 4–5 independent measurements, in each case normalised to the amount of HPRT transcript. The 1A and 1B forms of Irga6 were distinguished by 5′ primers specific respectively for exon 1A and exon 1B, while a common 3′ primer in exon 2 was used.
(1.24 MB TIF)

Figure S3 Western blots to assay Irgm3, Irgb6 and Irgm1 in lysates of tissue culture cells and primary hepatocytes. A. From left to right: Irgm3, assayed in L929 fibroblasts and TiB75 hepatocytes after (+) and without (−) stimulation for 24 hr with 100 U/ml IFNg; whole liver, and enriched primary hepatocytes cultured for 0, 3, 6, 12 and 24 h without IFNg, and lastly for 24 h with 100 U IFNg (24+). Calnexin was assayed in each sample as a loading control. B. From left to right, Irgb6 and Irgm1 assayed in TiB hepatocytes without (−) and with (+) IFNg stimulation for 24 hr with 100 U/ml; whole liver; enriched primary hepatocytes cultured for 0, 3 and 24 h without (−) and with (+) 100 U/ml IFNg. Calnexin was assayed in each sample as a loading control.
(0.38 MB TIF)
