# Supplementary material for: A Dedicated Promoter Drives Constitutive Expression of the Cell-Autonomous Immune Resistance GTPase, Irga6 (IIGP1) in Mouse Liver
Source: PLoS One. 2009 Aug 26;4(8):e6787. doi: 10.1371/journal.pone.0006787 (PMC2848866; doi:10.1371/journal.pone.0006787)
Supplement: Table S1 — Promoter HNF elements (0.03 MB DOC) [file pone.0006787.s001.doc]

**Supplementary Tables**

**Table S1**

Promoter HNF Elements in order 5’-> 3’ (see also Fig 4A and 4B)

HNF-1 **DGTTAATNATTAAH**

Irga6_1B **TGTTAATCATTCAA**

Irga1 **TGTTAATCATTGAA**

HNF-4 **AGGTCAN(N)AGG TC**

Irga6_1B **AGGTCAT G AGGCTC**

Irga1 **AGGTCAT G AAGGAC**

HNF-6 **WWATKGAYTT**

Irga6_1B **TTATGGACT**C

Irga1 **TTAT**A**GACT**C

HNF-6(-) **AARTCMATWW**

Irga6_1B **AAA**A**CAATCT**

Irga1 **AAA**A**CAATCT**

Irga2 **AAA**A**CAATCT**

HNF-4(-) **GACCTN(N)TGACCT**

Irga6_1B **GATCTT C TGACCT**

Irga1 **GATCTT C CAACCT**

Irga2 **GATCTT C TGACCT**

HNF-6(-) **AARTCMATWW**

Irga6_1B **AACACAATTT**

Irga1 **AACACAATTT**

Irga2 **AACACAATTT**

HNF-3ß **CAATATTTACTT**

Irga6_1B **CAATTTCTACTT**

Irga1 **CAATTTCTACTT**

Irga2 **CAATTTCTACTT**

The universal codes for specifying ambiguous bases are: R=A/G, Y=C/T, M=A/C, K=G/T, S=C/G, W=A/T, B=C/G/T, D=A/G/T, H=A/C/T, V=A/C/G, and N=A/C/G/T.

The putative promoter regions of Irga1 and Irga2 are homologous to the Exon 1B promoter of Irga6. Irga1 and Irga2 both possess canonical HNF binding sites clusters in their putative promoter regions (see also Fig 4A and 4B). The region of homology for Irga2 is truncated and only the 3’ cluster of HNF sites is conserved with Irga6.
