# Supplementary material for: A Dedicated Promoter Drives Constitutive Expression of the Cell-Autonomous Immune Resistance GTPase, Irga6 (IIGP1) in Mouse Liver
Source: PLoS One. 2009 Aug 26;4(8):e6787. doi: 10.1371/journal.pone.0006787 (PMC2848866; doi:10.1371/journal.pone.0006787)
Supplement: Table S2 — Directory of Irga gene sequence elements on chromosome 18 genomic contig, NT_039674.7 (0.06 MB DOC) [file pone.0006787.s002.doc]

**Table S2**

Directory of Irga gene sequence elements on chromosome 18 genomic contig, NT_039674.7 (See also Fig 5C)

| Gene | Element | bp 5’ Start* | bp 3’ End** |
| --- | --- | --- | --- |
| Irga1ψ | Exon 1 5’UT | 57,371,717 | 57,371,823 |
|  | Intron length bp | 8,676 | |
|  | Exon 2ψ (by homology) | 57,380,499 | 57,381,386 |
|  | Intergenic distance bp | 17,200 | |
| Irga2ψ | Exon 1ψ | 57,398,586 | 57,398,698 |
|  | Intron length bp | 6,351 | |
|  | exon 2 | 57,405,049 | 57,406,269 |
|  | Intergenic distance | 26,657 | |
| Irga3 | Exon 1 | 57,432,926 | 57,432,823 |
|  | Intron length | 3,506 | |
|  | Exon 2 | 57,430,673 | 57,429,420 |
|  | Intergenic distance | 22,623 | |
| Irga4 | Exon 1 | 57, 453,036 | 57,453,140 |
|  | Intron length | 6,364 | |
|  | Exon 2 | 57,459,504 | 57,460,751 |
|  | Intergenic distance | 23,741 | |
| Irga5ψ | Exon 2ψ (by homology) | 57,484,492 | 57,485,061 |
|  | Intergenic distance | 33,213 | |
| Irga7ψ | Exon 1 | 57,518,274 | 57,518,376 |
|  | Intron length | 2,721 | |
|  | Exon 2 | 57,521,097 | 57,522,363 |
|  | Intergenic distance | 13,317 | |
| Irga6 | Exon 1a | 57,535,680 | 57,535,784 |
|  | Distance Exon 1A-1B | 6,558 | |
|  | Exon 1b | 57,542,342 | 57,542,448 |
|  | Intron length | 7,018 | |
|  | Exon 2 | 57,549,466 | 57,550,707 |
|  | Intergenic distance | 48,341 | |
| Irga8 | Exon 1 | 57,599,048 | 57,599,152 |
|  | Intron length | 2,255 | |
|  | Exon 2 | 57,601,407 | 57,602,627 |

*Base pair numbers according to NT_039674.7. For 5’ bases of exon 1, this normally records the most upstream base confirmed in an EST, for 5’ bases of exon 2, this normally records the first bp after the splice acceptor signal. The unused hypothetical exon 1 of Irga2 was defined by homology with other exon 1B sequences.

**Base pair numbers according to NT_039674.7. For 3’ bases of exon 1, this records the last bp before the splice donor signal. For 3’ bases of exon 2, this records the last base of the termination codon: the 3’UT has not been considered. For pseudogenes with an interrupted reading frame, as Irga1 and Irga5, this refers to the stop codon defined by homology with other Irga genes.
