# Supplementary figures and images for: A Dedicated Promoter Drives Constitutive Expression of the Cell-Autonomous Immune Resistance GTPase, Irga6 (IIGP1) in Mouse Liver
Source: PLoS One. 2009 Aug 26;4(8):e6787. doi: 10.1371/journal.pone.0006787 (PMC2848866; doi:10.1371/journal.pone.0006787)

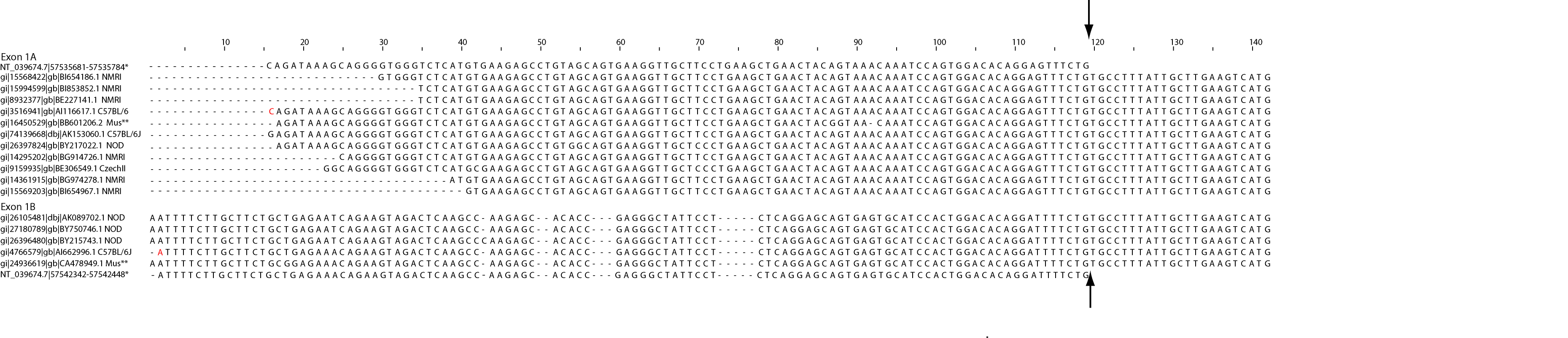

Supplement: Figure S1 — Mouse EST sequences corresponding to Irga6 were recovered from the public databases using Megablast. The figure shows an alignment of all ESTs with extended sequence in the 5′-untranslated region. The genomic regions corresponding to exon1A and exon 1B were identified from the C57BL/6 chromosome 18 contig, NT_039674.7, and are given at top and bottom of the alignments. The splice boundary between both exon 1A and exon 1B and exon 2 is indicated by arrows above and below the alignment. The last three bases of each EST sequence in the alignment form the initial start codon of the Irga6 translated protein. We used the 5′ nucleotide of the two C57BL/6-derived ESTs, AI116617 and AI662996, to define the start-point of transcription for exon 1A and exon 1B respectively. These correspond to nucleotide 57,535,681 of NT_039674.7 and for exon 1A and nucleotide 57,542342 for exon 1B (both printed in red). Non-genomic 5′ sequence extensions were removed from some EST sequences before alignment. A splice variant of Irga6 was described recently in IFN-induced mouse cells which carries both exon1A and exon 1B in tandem in that order, with a short linker sequence between them (NM_001146275) [16]. This is a unique case and undoubtedly should not be considered a normal splice variant. * indicates mouse strain of origin of EST. ** indicates ESTs from libraries where the Mus domesticus strain of origin was not recorded. (0.26 MB TIF) [file pone.0006787.s003.tif]

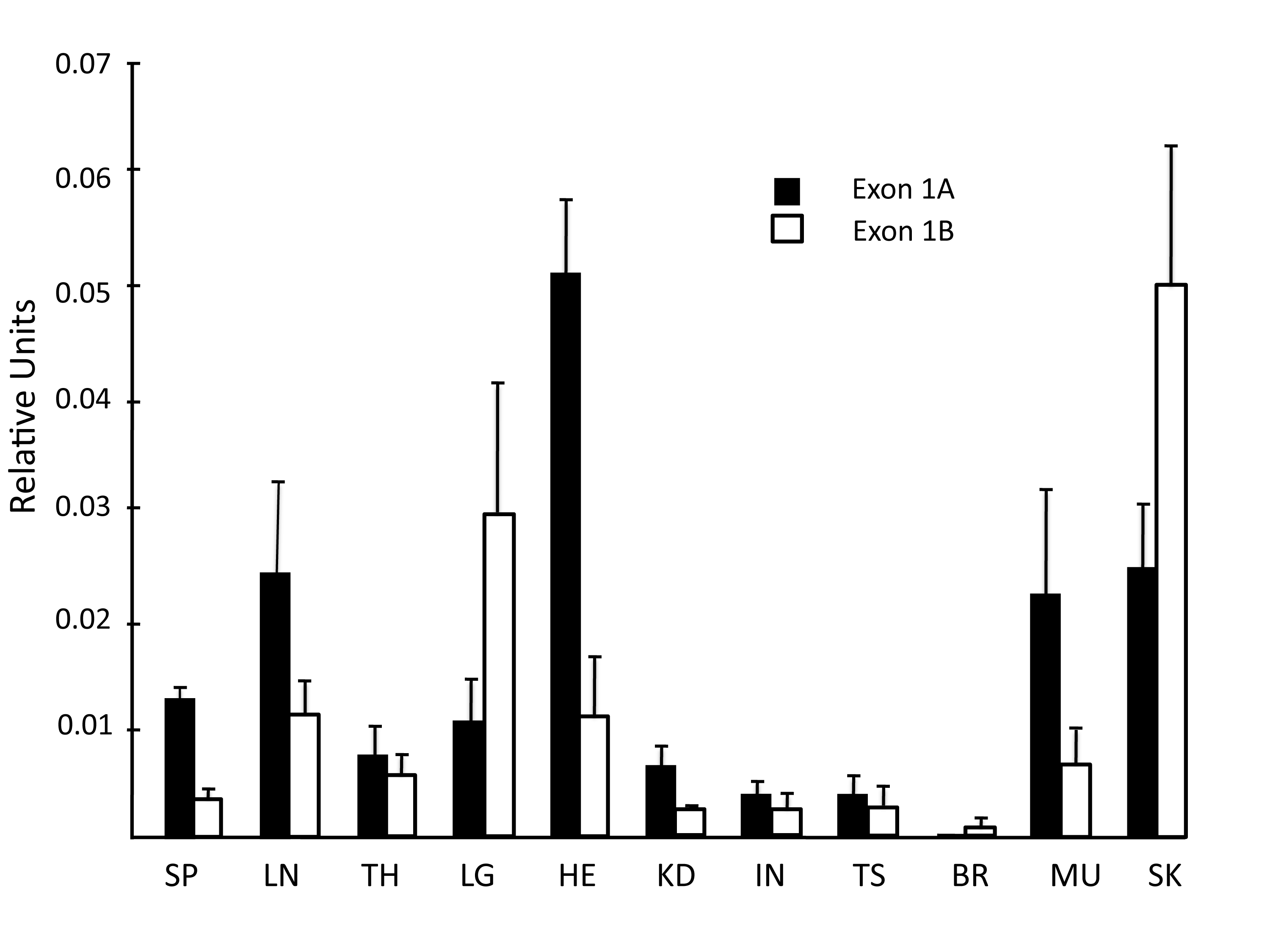

Supplement: Figure S2 — Real-time PCR to analyze Irga6 expression in RNA purified from different organs of adult C57/BL6 mice. Results are presented as means with standard deviations from 4–5 independent measurements, in each case normalised to the amount of HPRT transcript. The 1A and 1B forms of Irga6 were distinguished by 5′ primers specific respectively for exon 1A and exon 1B, while a common 3′ primer in exon 2 was used. (1.24 MB TIF) [file pone.0006787.s004.tif]

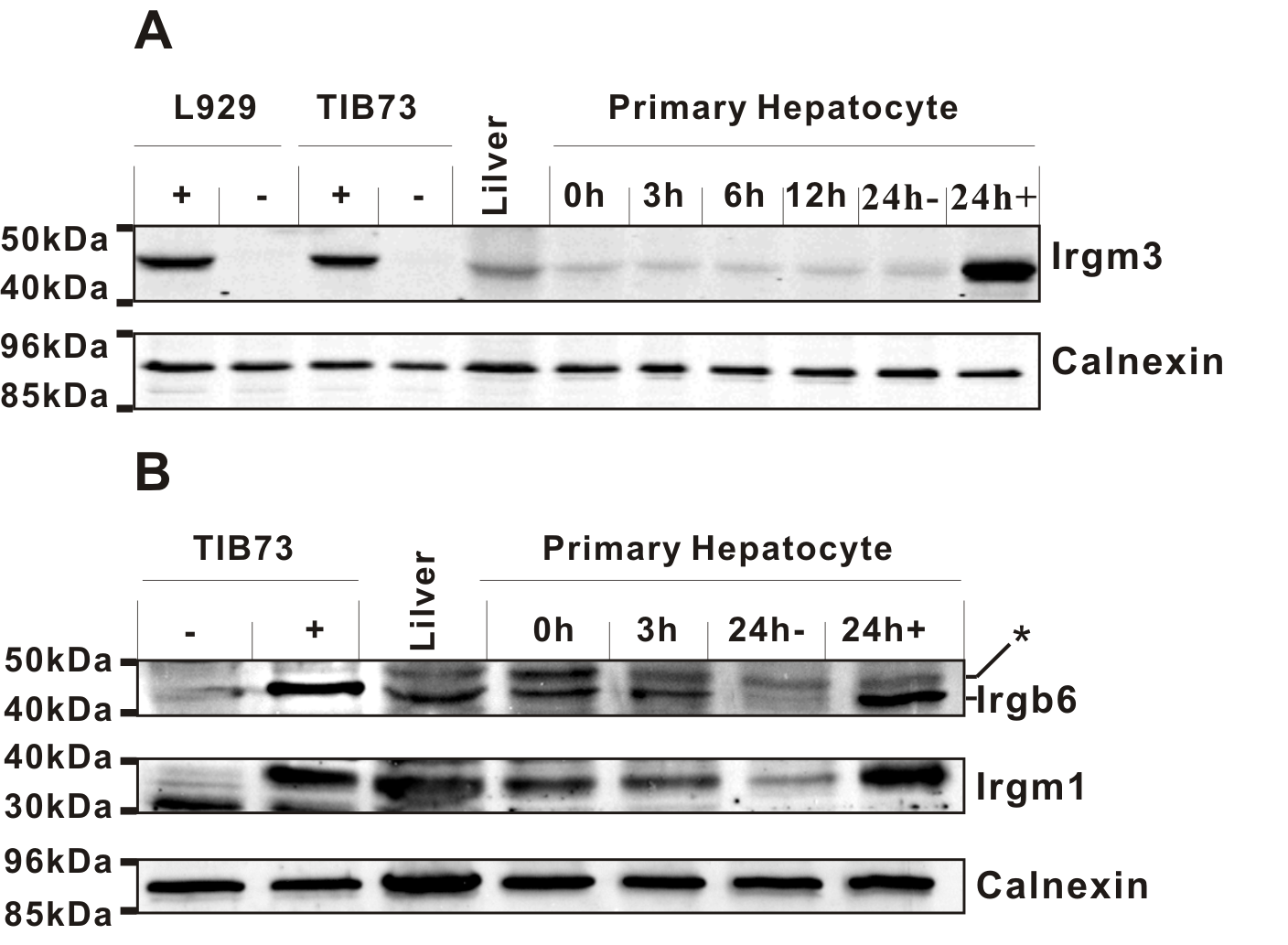

Supplement: Figure S3 — Western blots to assay Irgm3, Irgb6 and Irgm1 in lysates of tissue culture cells and primary hepatocytes. A. From left to right: Irgm3, assayed in L929 fibroblasts and TiB75 hepatocytes after (+) and without (−) stimulation for 24 hr with 100 U/ml IFNg; whole liver, and enriched primary hepatocytes cultured for 0, 3, 6, 12 and 24 h without IFNg, and lastly for 24 h with 100 U IFNg (24+). Calnexin was assayed in each sample as a loading control. B. From left to right, Irgb6 and Irgm1 assayed in TiB hepatocytes without (−) and with (+) IFNg stimulation for 24 hr with 100 U/ml; whole liver; enriched primary hepatocytes cultured for 0, 3 and 24 h without (−) and with (+) 100 U/ml IFNg. Calnexin was assayed in each sample as a loading control. (0.38 MB TIF) [file pone.0006787.s005.tif]
